# Contextual-Cueing beyond the Initial Field of View—A Virtual Reality Experiment

**DOI:** 10.3390/brainsci10070446

**Published:** 2020-07-13

**Authors:** Nico Marek, Stefan Pollmann

**Affiliations:** 1 Department of Psychology, Otto-von-Guericke Universität Magdeburg, 39106 Magdeburg, Germany; stefan.pollmann@ovgu.de; 2 Center for Brain and Behavioral Sciences, Otto-von-Guericke Universität Magdeburg, 39106 Magdeburg, Germany; 3 Beijing Key Laboratory of Learning and Cognition and School of Psychology, Capital Normal University, Beijing 100048, China

**Keywords:** visual search, contextual cueing, virtual reality

## Abstract

In visual search, participants can incidentally learn spatial target-distractor configurations, leading to shorter search times for repeated compared to novel configurations. Usually, this is tested within the limited visual field provided by a computer monitor. While contextual cueing is typically investigated on two-dimensional screens, we present for the first time an implementation of a classic contextual cueing task (search for a T-shape among L-shapes) in a three-dimensional virtual environment. This enabled us to test if the typical finding of incidental learning of repeated search configurations, manifested by shorter search times, would hold in a three-dimensional virtual reality (VR) environment. One specific aspect that was tested by combining virtual reality and contextual cueing was if contextual cueing would hold for targets outside the initial field of view (FOV), requiring head movements to be found. In keeping with two-dimensional search studies, reduced search times were observed after the first epoch and remained stable in the remaining experiment. Importantly, comparable search time reductions were observed for targets both within and outside of the initial FOV. The results show that a repeated distractors-only configuration in the initial FOV can guide search for target locations requiring a head movement to be seen.

## 1. Introduction

The ability to locate objects in a complex environment is both a demanding and crucial task in our everyday lives. Given the nature of our world, target objects do not appear isolated from other stimuli but within a complex environment that provides information to guide our attention as well as distracting objects. Frequently used items are very likely to be found in a spatial context among several other items (e.g., toothbrush in the mirror cabinet is placed next to the toothpaste, face cream, shaver and other cosmetic products). This configuration can provide information to guide visual search for a target item when the configuration is encountered again. Although we greatly benefit from this additional information, we may actually be unaware of these spatial cues. In the lab, this phenomenon is referred to as Contextual Cueing [1].

### 1.1. Contextual Cueing

The classical contextual cueing paradigm is a visual search task in which participants are instructed to search a T-shaped target among L-shaped distractors. Importantly, in half of the trials (repeated configuration) the spatial target-distractor configuration is repeated while in the other half the distractor configurations are generated randomly for each presentation (new configuration). Numerous studies have shown that search times are reduced over time for the repeated displays relative to the new displays. This reduction occurs although participants are not told about the repeated presentations and are usually not aware of it. Even if they become aware, the degree of awareness is not related to the size of search facilitation [2,3], so that the contextual cueing effect is mostly dependent on implicit learning [4]. The task of the participants is to indicate the (left or right) orientation of the tilted T-shaped target. The tilt of the target is random, so that no association between repeated target-distractor configurations and the response can be learned. This prevents that a response is elicited—e.g., based on a hunch that a configuration is familiar—before the target is actually found.

### 1.2. Contextual Cueing and Virtual Reality

When we search for an object, we often have to make head movements in order to find it. We want to investigate this situation in the present study. In prior studies of contextual cueing, the complete target-distractor configuration was presented within the field of view of the participants. Even in experiments that used projection systems to create somewhat larger displays [5], head movements were not necessarily used, because human observers can scan a field of view (FOV) of ca. 180°, using eye movements to bring more peripheral parts of the FOV into focus. Note that by using a virtual reality (VR) device, the initial FOV is more restricted. Locations outside of the initial FOV can only be brought into the FOV by moving the head. In the case of our search paradigm, this meant that in a portion of the repeated displays in which the target was placed outside of the initial FOV, search guidance had to depend on a distractors-only configuration in the initial FOV leading the way to the target location. Thus, one important question of the present study was: Is the repeated distractors-only configuration sufficient to facilitate the search for the target?

Here, we used virtual reality technology to address this question. Due to recent developments in display manufacturing binocular head mounted displays (HMDs) became affordable for many researchers. Compared to regular computer setups, HMDs have some advantages. Monitors for example usually do not require head movement and in order to investigate panorama-like search displays either (1) larger monitors must be purchased or (2) search displays themselves must be rescaled. Virtual reality (VR) on the other hand allows to create larger search displays that can be explored with head movements. In this context the first research question is straightforward: Is the contextual cueing effect replicable in a virtual reality environment? The second question was if participants would benefit from a repeated target-distractor configuration even if the initial field of view did not contain the target. To our knowledge, this is the first study to address this question.

## 2. Method

### 2.1. Participants

A total of 19 (11 females; eight males; M = 26.39; SD = 6.3) participants with normal or corrected to normal vision took part in this study. None of the participants had any previous experience with HMDs. The participants were from different backgrounds, including psychology students as well as regular workers. All participants were naïve to the experimental conditions and provided informed written consent prior to the experiment. The experiment was approved by the ethics committee of the Medical Faculty of the Otto-von-Guericke University Magdeburg (ethic code: Az 72/18).

### 2.2. Apparatus

The task was implemented in C# using the Unity engine (version 2019.1.0f2), OpenVR (version 1.1.3b) and the Virtual Reality Toolkit (version 3.2.0). A HTC Vive HMD was used for stimulus presentation and its respective controllers for response recording. Stimuli were presented on two displays (one per eye) with a diagonal size of 91.4 mm and a resolution of 1200 × 1080 pixels each. Although VR is often used to present near-realistic scenes, we designed a VR-version of the often-used T-among-L search to keep the search task as similar as possible to previous contextual cueing studies, except for its 3D-aspect.

### 2.3. Stimuli and Virtual Environment

**Figure 1:**
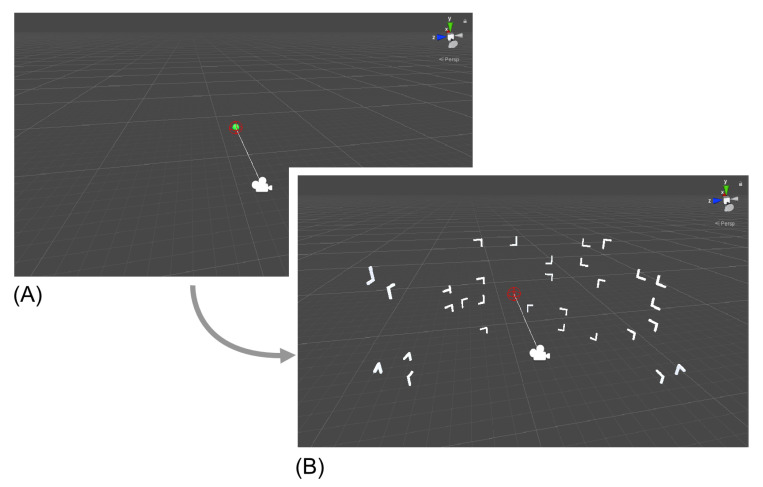
An overview from the “scene view” in Unity Engine. The camera icon is the visualization of the participants head. The red sphere and the corresponding vector indicate the orientation of the field of view (FOV) and were invisible for the participant. (**A**) Checking the head position with the help of a fixation object. If the red sphere aligned with the green fixation object a new trial was started. (**B**) An overview of an exemplary search display.

**Figure 2:**
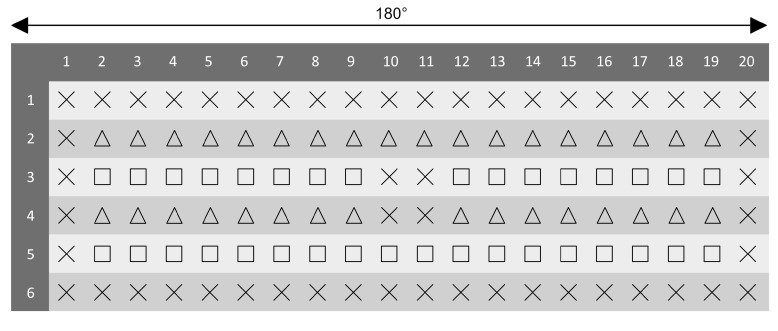
A two-dimensional overview of possible object spawning spanning the first 180° of each imaginary circle. There were three possible placement options in total: Distractor but no target (**X**), distractor and target for the new configuration (**triangle**) and distractor and target for the repeated configuration (**square**).

While previous two-dimensional studies placed target and distractors on a two-dimensional plane, in the present experiment all possible item positions were placed on a half-cylinder spanning the anterior 180° of a circle with the participant at its center (Figure 1B). Object size was unified via a scaling parameter of 0.4 units (around 3.8°× 3.8° of visual angle) and the distance between the center of the circles and object locations was fixed to six units (by default distances in Unity are measured in meters; 1 virtual unit = 1 m.). Each search display was composed of 1 target (90° or 270° rotated T) and 30 distractors (0°, 90°, 180°, 270° rotated L’s). Object placement was possible on 20 equidistant locations per circle. The gap between each circle was one virtual unit. All boundary locations (circles one and six, position one and 20 of the intermediate circles) or at locations in the center of each display (position 10 and 11 of circles three and four) were excluded as possible target locations in both conditions. Target placement in the new configuration was restricted to circles two and four and for the repeated configuration to circles three and five (see Figure 2). All object positions were counterbalanced across quadrants and conditions. A spherical object was placed in the center of each display as fixation mark in between trials.

The virtual environment was evenly illuminated by a virtual light source placed directly at the center of the imaginary circles. As background color, a dark blue was chosen (hex color: #314D7900) and a white shader (hex color: #FFFFFF00) was attached to every object. The entire project is available at https://github.com/nimarek/Contextual-Cueing-Unity. We also provide a tutorial for the implementation of Virtual Reality experiments in general and Contextual Cueing in particular [6].

### 2.4. Design and Procedure

Every experiment consisted of four successive phases. (1) HMD calibration and adjustments, (2) a short familiarization task, (3) the actual experiment and (4) a conclusive recognition task followed by a short questionnaire and debriefing. One session lasted approximately 1 h in total.

Each session started with individual adjustment of the distance between both HMD displays. Calibration of the room scale component was carried out using the SteamVR room setup module. The position of the lighthouse tracking cubes remained constant at 1.5 m above ground throughout the entire study. Participants were able to move in a designated area spanning 2.5 m × 2.5 m. If a participant came close to physical borders, a two-dimensional semi-transparent blue grid texture was displayed inside the VR environment. The grid disappeared after the participant returned to the center position.

After finishing initial adjustments, the experiment started with a familiarization task of 20 trials. Participants were informed that the upcoming task would contain fewer objects in total and that the main experiment would use larger search displays (see Figure 1B). Participants looked for a rotated T among L-shaped distractors and indicated the target’s orientation by pressing either the trigger button on the left or the right controller. In both the familiarization task and the main experiment a false response was detected if the input did not match the orientation of the target. Feedback about their accuracy was provided verbally. All display configurations of the familiarization task were completely randomized, containing no target positions that would be reused in the upcoming experimental blocks. Furthermore, the difficulty of each trial was reduced by limiting the number of possible distractor locations per circle to 10 and the actual presented distractors to two. The main search experiment was composed of 16 blocks with 32 trials each. At the beginning of each session eight repeated configurations were generated and saved. These trials were repeated twice within every block and intermixed with semi-randomly generated configurations (new configuration). The fixed FOV of 110° in combination with a fixed radius between every asset and participant ensured that only positions five to 15 were potentially visible without moving the head (near condition). Search outside this initial FOV (Target locations 2–4 and 16–19, see Figure 2) required head-movement (far condition). While the distractor placement and orientation remained constant for the repeated configuration, orientations of the target were randomly chosen at the beginning of every trial and did therefore not correlate with a specific condition. Trial order was randomized using a variation of the Fisher–Yates shuffle algorithm [7], followed by a subsequent repetition check. If the same trials were listed directly after another, the shuffle was executed again. All participants were allowed to have self-determined breaks after finishing each block.

Every trial started with a fixation object (see Figure 1A) in the center of each search display for 1500 ms. It gradually changed its color from red (0 ms) to green (1500 ms). Orientation of the FOV direction was furthermore checked via an invisible vector starting from the participants head coordinates with a length equal to the radius. This vector was moved along the *Z*-axis in the Unity coordinate system. As soon as this vector pointed to the fixation object and 1500 ms had passed, a new trial was started. This technique allowed us to guarantee a constant alignment of the initial FOV throughout all trials and thus ensured that targets outside the initial FOV were not visible to the participant. Search displays themselves remained present until the participant used one of the input buttons.

Once all experimental blocks were finished, the participants performed a concluding recognition task. Eight repeated configurations were mixed with eight new configurations. Displays were presented in randomized order. Subjects were asked whether they had seen the displays before or not. Yes was indicated by pressing the right controller button, no by pressing the left controller button. A conclusive questionnaire asked for potential technical problems, participants’ explicit knowledge about the repeated configuration, previous experience with computer gaming and VR experience in general.

### 2.5. Data Analysis and Data Exclusion

Data were analyzed via a custom-made R script (version 3.6.2) [8]. A mixed effects analysis of variance ANOVA was implemented using the Analysis of Factorial Experiments (afex, version 0.27-2) library Alpha level was set at 0.05 for all statistical tests. All reported effect sizes are generalized eta-squared effects [9]. Bayes factors were calculated using the BayesFactor R package (version 0.9.12-4.2). Figures were created using a custom-made Python script (version 3.7) and the seaborn library (https://seaborn.pydata.org/).

The following three successive steps were performed before data analysis: (1) trials containing a false response were excluded, (2) every trial containing a reaction time (RT) below 200 ms was excluded from the dataset and (3) all trials longer than 3.5 standard deviations from the average search time were removed from further analysis. In total 1.45% of all trials were removed from the dataset. Three participants’ data were excluded from the analysis—one because of technical difficulties, another two participants reported motion sickness. The subsequent analysis includes a total of 16 subjects.

### 2.6. Results

In order to increase statistical power, every four consecutive blocks were aggregated into a single epoch, resulting in four epochs overall. Search accuracy was high, ranging from 96.31% to 100% (average 98.71%). To rule out potential speed-accuracy trade-offs, we have analyzed accuracy (Table 1) by mean of a repeated measures analysis of deviance on a logistic regression model of the error frequency, with configuration (repeated and new), target position (far and near) and epoch (epochs 1–4) as factors. The analysis did not yield any significant main effects or interactions (all p>0.05).

**Figure 3:**
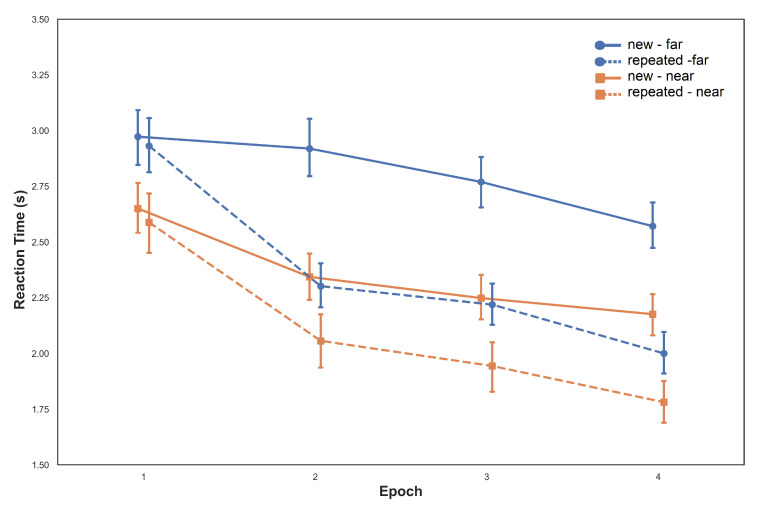
Mean reaction times as a function of epoch and associated standard errors for both repeated and new configurations. Solid lines depict new configurations, dashed lines depict repeated configurations. Blue lines indicate trials with the target outside of the initial FOV (far), orange lines indicate trials with the target inside the initial FOV (near).

A repeated-measures ANOVA with configuration (repeated and new), epoch (1–4) and target position (far and near) as factors was performed to investigate mean RTs. Analysis revealed a significant main effect of epoch $\left[F\left(1,45\right)=16.28,p<0.001,\eta^{2}G=0.09\right]$. Participants searched faster as the experiment progressed. A significant main effect for configuration $\left[F\left(1,\;15\right)\;=\;8.04,p\;<\;0.05,\eta^{2}G\;=\;0.03\right]$ was also observed with shorter reaction times for repeated configurations. A significant main effect for target position $\left[F\left(1,15\right)=9.83,p\;=\;0.007,\eta^{2}G\;=\;0.08\right]$, indicated that participants reaction times were larger when the target was placed outside of the initial FOV, this is illustrated in Figure 3. The interaction of epoch and configuration $\left[F\left(3,\;45\right)\;=\;10.99,p\;<\;0.001,\eta^{2}G=0.01\right]$ was also significant, reflecting that the magnitude of contextual cueing effects increased across epochs. This describes the time advantage in the repeated configuration relative to the new configuration. The interaction of configuration and target position was not significant $\left[F\left(3,15\right)<1,p=0.96,\eta^{2}G<0.0001\right]$. This non-significant interaction may be due to comparable size of contextual cueing effects for target positions inside the initial FOV and outside of it. However, because the interaction does not inform us about the likelihood that two effects are of comparable size, we additionally calculated a Bayesian ANOVA. In a Bayesian framework, a Bayes Factor (BF) can indicate how well a hypothesis (here: H0 over H1) predicts the empirical data. The interaction of configuration x target position yielded a BF_01_ = 11.86, supporting the equality of contextual cueing effects for target in and out of the initial FOV. The interaction between epoch and target position $\left[F\left(3,45\right)<1,p=0.52,\eta^{2}G<0.0001\right]$ and the three-way interaction between epoch, configuration and target position $\left[F\left(3,45\right)<1,p=0.75,\eta^{2}G=0.0002\right]$ were also not significant.

The overall number of far trials (3861) was somewhat less than the number of near trials (4212). This imbalance was the result of the technical requirements of the hard- and software interacting with the search display layout and led to a more conservative test of the contextual cueing effect in the far condition.

### 2.7. Recognition Task

The subsequent recognition task included all eight repeated configurations as well as eight new configurations. If a repeated configuration was classified as known, it counted as a hit. If a new configuration was incorrectly classified as known, it counted as a false alarm. Participants mean hit rate was: 54.69% (SD = 13.84), their false alarm was 51.88% (SD = 11.96). Both the hit and the false alarm rate did not differ significantly from each other: t(15)=1.0259, p=0.3229. A BF01 = 1647 provided only anecdotal evidence for H0, reflecting the necessarily low statistical power of recognition tests that were limited by the number of repeated displays in the main experiment [10].

## 3. Discussion

The present study replicated contextual cueing in a virtual environment with panorama-like display sizes and target positioning outside of the initial FOV. Participants searched faster in repeated displays in a VR version of the classical T among L search task, replicating previous work with two-dimensional displays. Importantly, search facilitation was comparable for targets within and outside of the initial field of view, demonstrating search facilitation for target locations that can only be viewed after a head movement.

The current VR implementation of a classic contextual cueing experiment extends the findings of Chun and Jiang [1] in various ways. It incorporates the ability to freely explore a search display, which raises the ecological validity of the experiment in general. Contextual cueing effects remained stable throughout the experiment both for targets in and outside of the initial FOV. Thus, the presentation of a distractors-alone configuration in the initial FOV was sufficient to guide search for the far targets in repeated trials, although the number of far trials was somewhat less than the number of near trials.

Our findings resemble to some degree the study by Beesley et al. [11], who demonstrated that contextual cueing does not only rely on the relationship between target and distractors, but also on learned distractor-distractor configurations. In the present study no target-absent trials were used—as in Beesley et al. [11]—but in our “far” displays, participants were exposed to a distractors-only configuration in the initial field of view and could find the target only after a head movement. Thus, one possibility would be that our participants learned to associate the distractor configuration within the initial FOV with the target-distractor configuration after a head movement. However, it may also be that participants learn a single integrated spatial configuration across head movements, similar to what has been suggested for eye movements [12]. In any case, the repeated distractor configurations alone provided enough information to guide visual search to target locations outside the initial FOV.

Previous findings using eye tracking devices suggest that in the repeated configurations fewer fixations are needed and saccades approach the target in a more efficient way ([13,14]). The present study suggests that visual search can also guide head movements. Future experiments should focus on the intertwined analysis of head-movements and reaction times. It seems plausible that initial repeated distractor combinations either (1) increase the probability of initial head motion towards the target, compared to random head movements in the new configurations or/and (2) lead to earlier head movements towards the target. Unfortunately, due to technical problems we could not record head movement data to address this question.

Since only eight repeated configurations could be presented in the recognition task and the subsequent Bayes Factor analysis did not show a clear result, we do not want to draw conclusions about the implicit or explicit nature of contextual cueing.

We decided to use a symbolic T-among-L search task in order to enhance comparability with the many studies that used two-dimensional versions of this paradigm. However, VR technology is particularly suited to create immersive scenes. Future work may investigate if contextual cueing in such realistic scenes compares with the findings obtained in two-dimensional realistic scenes (e.g., [13,15]). In addition to the possibilities of creating more complex virtual environments, external factors can also be controlled better than in conventional experimental settings. The distance between the displays and the eyes remains constant across participants and room lighting has no influence on the test. Compared to other studies with larger search displays ([5]), search times have increased. However, the scope of the search displays used here cannot be reached by monitors or projectors. Accuracy does not seem to be affected by the VR technology.

In future studies of contextual cueing in an VR environment, the additional use of eyetracking data might be considered. The majority of studies on contextual cueing in two-dimensional did not analyze eye movement data, but relied on response times. This is what we have replicated for the VR environment in the present study. Measurement of eye movements, however, might be particularly useful if specific questions, like implicit reorienting of attention after changes of the target-distractor configuration [14] or the effects of vision loss on contextual cueing ([16,17,18]) are to be investigated.

## Figures and Tables

**Table 1:** Percentage of correct answers per epoch for all configuration and trial types.

	Epoch 1	Epoch 2	Epoch 3	Epoch 4
Overall	98.19%	98.97%	99.07%	98.38%
New Configuration	98.34%	99.41%	99.32%	99.12%
Repeated Configuration	98.05%	98.54%	98.83%	98.54%
Near Trials	97.46%	99.02%	98.83%	98.54%
Far Trials	98.93%	98.93%	99.32%	99.12%
